# Prediction Models for Postoperative Atrial Fibrillation After Cardiac Surgery: A Systematic Review and Critical Appraisal

**DOI:** 10.3390/jcm15135255

**Published:** 2026-07-05

**Authors:** Bryam López Tuesta, Yerson Alberca-Naira, Jhair Alexander Leon-Rodriguez, Jonathan Rodriguez-Pratto, Jose D. Andrade-Saavedra, Franck J. Calderon-Chilet, Carlos A. Sarmiento-Maldonado, Oriana Rivera-Lozada, Cesar Bonilla-Asalde, Joshuan J. Barboza

**Affiliations:** 1 Departamento de Cardiologia, Hospital Nacional Edgardo Rebagliati Martins, Lima 15073, Peru; blt138@hotmail.com; 2 Escuela de Medicina, Universidad Nacional de Piura, Piura 20002, Peru; yerson210918@gmail.com; 3 Universidad Cesar Vallejo, Trujillo 13001, Peru; jhleonr@ucvvirtual.edu.pe; 4 Facultad de Medicina, Universidad Del Norte, Barranquilla 080001, Colombia; jjrpratto@gmail.com; 5 Facultad de Medicina Humana, Universidad Privada Antenor Orrego, Trujillo 13008, Peru; jandrades1@upao.edu.pe; 6 Facultad de Medicina, Universidad Nacional Mayor de San Marcos, Lima 15081, Peru; franckjohancc@gmail.com; 7 Escuela Superior de Medicina, Instituto Politécnico Nacional, Mexico City 11340, Mexico; carlos.sm2802@gmail.com; 8 Escuela de Medicina, Universidad Señor de Sipan, Chiclayo 14002, Peru; riveraoriana@uss.edu.pe (O.R.-L.); bonasal@gmail.com (C.B.-A.)

**Keywords:** postoperative atrial fibrillation, prognostic factors, cardiac surgery, coronary artery bypass grafting, heart valve surgery, prediction models, meta-analysis, risk stratification, echocardiography, biomarkers

## Abstract

**Background/Objectives:** Postoperative atrial fibrillation (POAF) is a frequent complication after cardiac surgery and is associated with increased morbidity, prolonged hospitalization, and higher healthcare costs. Numerous multivariable prediction models have been developed to estimate individual risk; however, their methodological robustness, validation status, and clinical transportability remain uncertain. This systematic review aimed to critically evaluate the methodological quality, validation strategies, and predictive performance of multivariable prediction models developed to estimate the risk of postoperative atrial fibrillation (POAF) after cardiac surgery. **Methods**: In accordance with PRISMA 2020 guidelines, we conducted a comprehensive search of PubMed, Scopus, Web of Science, and Embase from inception to July 2025. Studies that developed or externally validated multivariable prediction models for POAF in adult patients undergoing cardiac surgery were eligible. Data extraction was performed using the CHARMS checklist, and methodological quality was assessed with PROBAST. Model performance was summarized descriptively, focusing on discrimination (C-statistic/AUC), calibration reporting, and validation strategies. **Results**: A total of 39 studies were included. Most models were based on logistic regression, whereas a minority employed Cox regression or machine learning techniques. Reported discrimination ranged from 0.60 to 0.98, demonstrating substantial heterogeneity in predictive performance. Calibration was inconsistently reported. Six studies performed external validation. According to PROBAST, 32 of 39 studies (82%) were rated at high risk of bias, predominantly within the analysis domain due to inadequate handling of overfitting, insufficient events-per-variable ratios, and limited validation procedures. **Conclusions**: Existing prediction models for POAF show variable discrimination but are frequently limited by high risk of bias, inadequate validation, and incomplete calibration assessment, thereby restricting their clinical applicability. Future research should prioritize rigorous external validation, transparent reporting in accordance with TRIPOD recommendations, and methodological strategies that enhance model generalizability and transportability across diverse surgical populations.

## 1. Introduction

Postoperative atrial fibrillation (POAF) is a prevalent, costly, and possibly detrimental complication subsequent to heart surgery. Different studies say that POAF happens between 20% and 40% of the time [1]. POAF is an independent factor that predicts various negative outcomes, including a 2- to 4-fold higher risk of stroke, reoperation due to bleeding, infection, kidney or lung failure, cardiac arrest, brain complications, the necessity for permanent pacemaker implantation, and a twofold increase in mortality from any cause at 30 days and 6 months [2]. The exact causes are not entirely known, but they include events that happen before and after surgery, such as inflammation, sympathetic activation, and cardiac ischemia, which all work together to cause atrial fibrillation, often in people who already have certain diseases [1].

Age, the type of cardiac surgery, myocardial ischemia, atrial enlargement, and pre-existing heart diseases are all possible risk factors that have been found. Additional contributing elements that induce sympathetic activation due to surgical stress include hypovolemia, intraoperative hypotension, anemia, and pain [3]. Unfortunately, because we do not know enough about how POAF happens after heart surgery, we have not been able to come up with any clear ways to stop or treat it. Nevertheless, the early identification of risk factors in the preoperative phase may contribute to the reductions in elevated morbidity, mortality, and treatment cost associated with POAF [4]. The current systematic study seeks to deliver a thorough and meticulous synthesis of prognostic markers related to POAF in adult patients having coronary, valvular, or congenital heart surgery.

## 2. Materials and Methods

### 2.1. Research Question

What prediction models have been developed or validated to estimate the risk of postoperative atrial fibrillation in adult patients undergoing cardiac surgery, and what is their methodological quality and predictive performance?

The research question follows the PICO framework, where the population of interest includes adults undergoing cardiac surgery, the prognostic factors consist of clinical, biochemical, imaging, and intraoperative variables, and the outcome of interest is the incidence of POAF diagnosed by electrocardiographic criteria.

### 2.2. Study Design

This investigation was carried out as a systematic review in accordance with the Preferred Reporting Items for Systematic Reviews and Meta-Analyses (PRISMA) standards. The study will adhere to the Transparent Reporting of a Multivariable Prediction Model for Individual Prognosis or Diagnosis (TRIPOD) framework to guarantee a systematic evaluation of predictive models. To improve openness and scientific rigor, the protocol will be registered in the International Prospective Register of Systematic Reviews (PROSPERO: CRD420251150234) before the selection of studies.

### 2.3. Eligibility Criteria

Studies were included if they evaluated adult patients undergoing coronary, valvular, or congenital cardiac surgery and assessed prognostic factors for POAF. Eligible studies must report on multivariable predictive models, including logistic regression, Cox proportional hazards models, machine learning algorithms, or other statistical techniques designed to evaluate risk stratification. Studies must provide adjusted effect estimates for prognostic factors associated with incident POAF. Prospective and retrospective cohort studies were included, while case reports, editorials, and studies focusing solely on treatment or prevention strategies were excluded.

Candidate predictors were categorized according to their temporal relationship to surgery into three predefined groups: (1) preoperative variables (e.g., demographic characteristics, comorbidities, echocardiographic parameters, biomarkers); (2) intraoperative variables (e.g., cardiopulmonary bypass duration, type of procedure); (3) early postoperative variables (e.g., inflammatory markers, immediate complications). This hierarchical classification allowed structured synthesis and avoided treating temporally distinct predictors as analytically equivalent.

### 2.4. Search Strategy

A thorough literature search was performed utilizing four principal electronic databases: PubMed, Embase, Scopus, and Web of Science. The search technique utilized a blend of Medical Subject Heading (MeSH) phrases and unstructured keywords associated with POAF, cardiac surgery, risk assessment, and prognostic modeling. We employed Boolean operators to make the search strategy better, making sure that sensitivity and specificity were balanced. To improve comprehensiveness, the reference lists of the included studies were manually reviewed, and citation tracking was conducted.

No restriction was applied regarding oncologic comorbidity status, allowing inclusion of studies evaluating malignancy as a predictor of POAF.

### 2.5. Study Selection Process

There were three steps in the process of choosing the studies. To begin, duplicate records were deleted in Rayyan web (https://www.rayyan.ai, accessed in 11 March 2026). Second, two reviewers who were not involved in the study looked at the titles and abstracts to find studies that met the qualifying requirements. Third, full-text screening was performed for papers that might be useful, and any differences were settled by discussion or by a third expert reviewer. A PRISMA flow diagram recorded the selection process to guarantee clarity and replicability.

Key prognostic factors related to POAF were identified based on provided effect size estimates (odds ratios [ORs], hazard ratios [HRs], or relative risks [RRs]) or allowed their calculation: PA-TDI duration (total atrial conduction time derived from P-wave onset (lead II) to the peak A’-wave on tissue Doppler imaging) ms, deceleration time (DT), revascularization of the ramus medianus, age, PR-interval ms, QRS-duration ms, LAE (left atrial enlargement), P wave > 120 ms lead II or terminal negative mode P-wave in V1 more than 40 ms wide and more than 1 mm deep, degenerative etiology, patients with ≥4 grafts, Hypokalemia (<3.6 mmol/L), prior episodes of AF, mitral valve regurgitation, aortic valve regurgitation, CHA2DS2-VASc (≥1 men or ≥2 women), heart failure, hypertension, acute coronary syndrome, ventricular tachycardia, statin use, CABG (coronary artery bypass graft), valve surgery, two surgical procedures, three surgical procedures, other ethnic group than white or black, surgical year from 2021, lower epicardial adiponectin, CABG + valve surgery, cardiac function (NYHA), LADi mm/m^2^ (Left atrial diameter index), LVEF (Left ventricular ejection fraction), preoperative-HbA1c, preoperative-STB (Serum total bilirubin), cardiopulmonary bypass time (CPB) min, operative time, postoperative WBC 10 9/L (white blood cell count), Neutrophil-to-lymphocyte ratio, creatinine, postoperative-creatinine, postoperative-BUN, postoperative-platelet count, postoperative-neutrophil count, postoperative-fever, white race, preexisting lung disease mild, preexisting lung disease moderate/severe, GFR < 90 mL/min, cardiogenic shock, preoperative aspirin, number of diseased coronaries, LAD mm (Left atrial diameter), LAD > 4.5 cm, Euro SCORE (per point), DFA1 (short-term scaling exponent of detrended fluctuation analysis), mitral Valve surgery, aortic Valve surgery, minimally invasive surgery, PAH (pulmonary arterial hypertension), CKD (Chronic Kidney disease), obesity, COPD (Chronic Obstructive Pulmonary Disease), BMI (m/kg^2^) (Body Mass Index), P duration (ms), A’ velocity (m/s), LVMI (left ventricular mass index) g/m^2^, LAVI ml/m^2^ (Left atrial volume index), ventilator Time > 8 h, CK-MB > 70 (ug/L) day 1, surgical time > 4 h, SII (systemic immune-inflammation index) > 545 × 10^9^/L, Euro SCORE II, reintervention, advanced aged (>65), rheumatic VHD (valvular heart disease), mitral stenosis, post-LVEF, postoperative LAVI, post MDPG (mean diastolic pressure gradient) of mitral valve, preoperative TR (tricuspid valve regurgitation), postoperative TR (tricuspid valve regurgitation), postoperative PAP (pulmonary artery pressure), levosimendan, PALS% (peak Atrial longitudinal strain), more than 1 cardiac surgical procedure, diastolic function grade 1, diastolic function grade 2, diastolic function grade 3, age, age > 60 a, male sex, ESM-1, ng/mL (endothelial cell–specific molecule), NT-proBNP pg/mL, BNP pg/mL, Urea, MPV, mean platelet volume, drinking history, PCWP (pulmonary capillary wedge pressure), PSAP (pulmonary artery systolic pressure), CI L/min/m^2^ (Cardiac index), use of beta-blockers, High-risk OSAHS (Obstructive sleep apnea/hypopnea syndrome), Cross-clamp time, Procalcitonin, Interleukin 6, SBP mmHg (Systolic blood pressure), Smoking, LA (Left atrial) reservoir strain, Diabetes, Dyslipidemia, and RA (right auricular) reservoir strain.

### 2.6. Outcomes

The primary outcome of interest was the incidence of postoperative atrial fibrillation (POAF), defined as a new-onset atrial arrhythmia occurring after cardiac surgery and documented by a standard 12-lead electrocardiogram or continuous telemetry monitoring. In most included studies, POAF was characterized by an irregular rhythm without discernible P waves, lasting at least 30 s or requiring medical intervention, including pharmacological treatment, electrical cardioversion, or modification of postoperative care.

Additionally, inflammatory and perioperative stress-related variables were considered in the synthesis when reported as independent predictors.

Whenever available, studies reporting longer monitoring periods (e.g., Holter monitoring or implantable loop recorders) were included, although the majority of studies limited follow-up to the in-hospital period or the first postoperative week. Secondary outcome definitions varied across studies but typically encompassed persistent or recurrent POAF, late-onset atrial fibrillation beyond hospital discharge, and associations of POAF with adverse clinical events such as stroke, reoperation, infection, renal or pulmonary failure, or all-cause mortality. For the purpose of meta-analysis, only studies reporting adjusted effect estimates for prognostic factors associated with incident POAF were synthesized.

Variability in POAF definitions and monitoring strategies across studies was extracted and descriptively synthesized.

### 2.7. Data Extraction

We used a standardized framework based on the CHARMS (Checklist for Appraisal and Data Extraction for Systematic Reviews of Prediction Modeling Studies) technique to get the data. The extracted data encompassed study parameters (author, year, country, study design, sample size), patient demographics, type of cardiac surgery, evaluated prognostic factors, model attributes, statistical methods, performance metrics, and external validation status. The management of absent data and any documented assessments of clinical impact were recorded.

### 2.8. Risk of Bias Assessment

The Prediction Model Risk of Bias Assessment Tool (PROBAST) was used to look at the risk of bias in the studies that were included. This tool looks at four important areas: participants, predictors, results, and statistical analysis. Studies were categorized as having low, moderate, or high risk of bias according to PROBAST score standards. The assessment also took into account other factors, such as how to deal with overfitting and if the model calibration methods were adequate.

Risk-of-bias judgments were conducted at the domain level (Participants, Predictors, Outcome, Analysis) according to PROBAST guidance. No numerical scoring system was applied. An overall risk-of-bias rating was determined following PROBAST recommendations, whereby a study was considered at high risk if at least one domain was judged as high risk.

### 2.9. Synthesis of Results and Data Analysis

Given the substantial clinical and methodological heterogeneity across the included studies, a quantitative meta-analysis was not performed. The identified models differed considerably in outcome definitions, predictor selection strategies, modeling techniques, validation procedures, and performance reporting. In light of this variability, a structured descriptive synthesis was conducted to ensure conceptual and methodological coherence with the objective of evaluating multivariable prediction models rather than isolated prognostic factors.

The synthesis focused on comparing model development characteristics, including study design, population features, modeling approaches, number and type of predictors retained in the final model, and methods used for handling continuous variables and missing data. Particular attention was given to internal validation strategies, such as bootstrapping or cross-validation, and to the presence and methodological rigor of external validation procedures, in order to distinguish apparent model performance from validated predictive accuracy.

Predictive performance was summarized descriptively, emphasizing discrimination metrics, primarily the C-statistic or area under the receiver operating characteristic curve (AUC), as well as calibration assessment when reported. Calibration was evaluated according to the methods described in each study, including calibration plots, Hosmer–Lemeshow testing, calibration slopes, or related approaches. Additional considerations included reporting transparency, adequacy of the events-per-variable ratio, and the potential for overfitting, in alignment with PROBAST methodological domains.

No pooling of heterogeneous effect estimates or frequency-based ranking of individual predictors was conducted, as the purpose of this review was to critically appraise prediction models as integrated multivariable constructs. This approach ensured that the analysis remained consistent with established methodological standards for systematic reviews of prognostic prediction models.

## 3. Results

### 3.1. Selection of Studies

**Figure 1:**
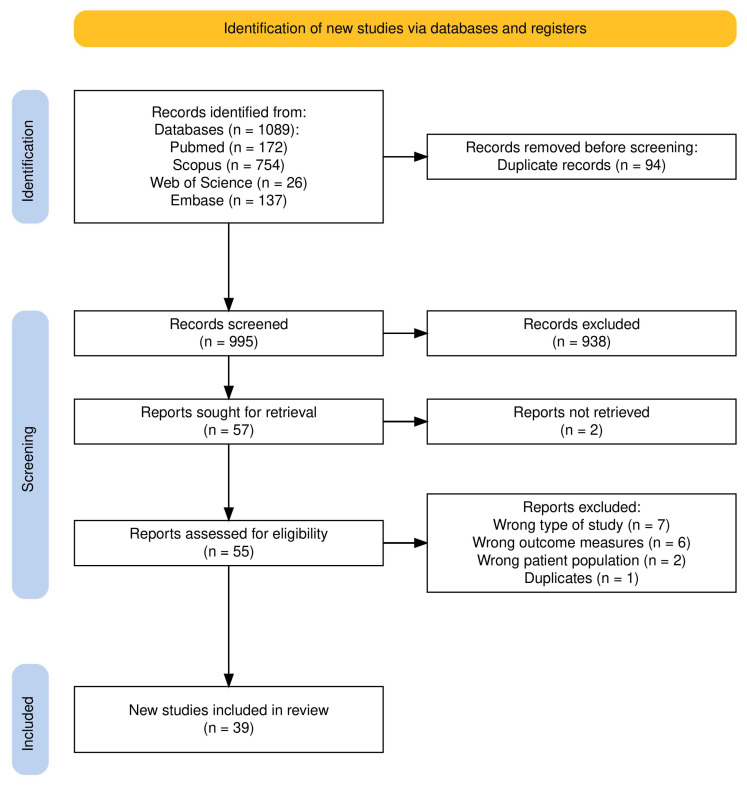
PRISMA 2020 Flow chart.

The initial search strategy across PubMed, Embase, Scopus, and Web of Science yielded a total of 1089 records. After the removal of 94 duplicates, 995 unique records were subjected to title and abstract screening. Of these, 938 were excluded as they did not meet the predefined eligibility criteria. A total of 57 full-text articles were retrieved for detailed evaluation, although two reports could not be obtained. Consequently, 55 studies underwent full-text review. Sixteen studies were excluded at this stage due to reasons such as wrong study design (n = 7), inadequate outcome reporting (n = 6), non-eligible patient populations (n = 2), and one duplicate publication. Ultimately, 39 studies fulfilled all inclusion criteria and were incorporated into the systematic review (5–43). The full process of study identification, screening, eligibility assessment, and final inclusion is summarized in Figure 1.

### 3.2. Characteristics of Included Studies

The characteristics of the studies included in this review are summarized in Table 1. Overall, the majority of studies evaluated prognostic factors and prediction models for the development of postoperative atrial fibrillation (POAF) in adult patients undergoing cardiac surgery. As shown in Table 1, the populations varied considerably in terms of sample size, ranging from fewer than 100 participants in some single-center prospective cohorts to several thousand in large registry-based studies. Surgical settings included isolated coronary artery bypass grafting (CABG), valve procedures, and combined surgeries, with most studies focusing on patients in sinus rhythm at baseline and excluding those with persistent preoperative atrial fibrillation. Follow-up was generally limited to the in-hospital period or the first postoperative week, although a subset of studies extended monitoring to several months or even years using implantable loop recorders.

Regarding outcomes and predictors, most studies employed a standardized definition of POAF, typically as new-onset atrial fibrillation confirmed by electrocardiography or continuous telemetry, lasting from at least 30 s to several minutes, and occurring during hospitalization. Candidate predictors encompassed a wide spectrum of variables, including demographic characteristics (age, sex, race, body mass index), comorbidities (hypertension, diabetes, heart failure, chronic obstructive pulmonary disease), clinical parameters (blood pressure, heart rate, New York Heart Association functional class), echocardiographic indices (left atrial diameter, left atrial volume index, diastolic dysfunction grade), electrocardiographic markers (PR interval, P-wave morphology, heart rate variability), laboratory biomarkers (C-reactive protein, interleukin-6, NT-proBNP, renal function markers), surgical characteristics (type of surgery, cardiopulmonary bypass and cross-clamp times), and novel omics-based markers such as gene expression and DNA methylation signatures. The number of predictors considered per study ranged from fewer than ten in focused analyses to more than two hundred in large-scale models, although the final multivariable models usually retained a small set of independent predictors. Frequently reported predictors of POAF included advanced age, previous atrial fibrillation, left atrial enlargement, impaired atrial strain, longer operative time, and elevated postoperative inflammatory markers.

Logistic regression was the most commonly applied modeling strategy, although some studies employed Cox proportional hazards models, penalized regression techniques such as LASSO, or machine learning approaches including random forest. Predictor selection was often based on a combination of clinical plausibility and univariate statistical significance, with stepwise forward or backward procedures commonly used. Internal validation was inconsistently performed, most often through bootstrap resampling, cross-validation, or data splitting, while only a minority of studies conducted external validation in independent cohorts. Measures of discrimination varied, with reported C-statistics ranging from 0.60 to 0.98, reflecting a broad spectrum of predictive performance across studies. Calibration was less frequently reported, but when assessed, it was generally adequate, with Hosmer–Lemeshow tests and calibration plots supporting acceptable model fit. Some models were presented in user-friendly formats, including nomograms and risk scores such as the PAFAC and POLARIS scores, designed to facilitate bedside clinical application.

### 3.3. Risk of Bias and Applicability Assessment

According to PROBAST domain-based assessment, 27 of 39 studies (69%) were rated at high overall risk of bias. The Analysis domain contributed most frequently to high-risk judgments, primarily due to inadequate events-per-variable ratios, absence of appropriate internal validation procedures, and insufficient reporting of model calibration. In several studies, insufficient events-per-variable ratios were either explicitly reported or could be inferred from the sample size and number of predictors included, suggesting a substantial risk of overfitting. Missing data handling was incompletely reported in many studies; when described, complete-case analysis was the most common approach, whereas multiple imputation was rarely implemented. Calibration assessment was inconsistently performed, with some studies relying solely on Hosmer–Lemeshow testing, whereas calibration plots and slope/intercept reporting were uncommon. External validation was performed in six studies and was clearly distinguished from internal validation methods such as bootstrapping or cross-validation (Supplementary Table S1).

### 3.4. Patterns of Predictor Inclusion Across Multivariable Models

Preoperative predictors were the most consistently incorporated variables across models, including age, left atrial structural parameters, and baseline comorbidities. Across the included studies, a wide spectrum of candidate predictors was incorporated into multivariable models for postoperative atrial fibrillation. Age was the most consistently retained predictor, appearing in the majority of final model specifications, followed by structural atrial parameters such as left atrial volume index.

Intraoperative variables, particularly cardiopulmonary bypass duration and procedural complexity, were incorporated in a subset of models aiming to refine perioperative risk estimation. Inflammatory and perioperative stress-related biomarkers, including systemic immune-inflammation index and interleukin-6, were incorporated in a limited number of models but demonstrated relevance within those specific modeling frameworks. Perioperative variables, such as postoperative fever and inflammatory cell counts, were included in certain models, although their incorporation varied depending on whether the objective was preoperative or perioperative risk stratification.

Early postoperative variables, such as inflammatory markers or immediate clinical complications, were included in certain dynamic prediction models; however, these predictors may limit applicability for purely preoperative risk stratification. Conversely, traditional cardiovascular risk factors such as smoking status, diabetes, and dyslipidemia were inconsistently retained across models, suggesting limited incremental predictive value in multivariable contexts. Electrocardiographic markers were occasionally incorporated but lacked uniform validation.

The marked variability in predictor selection strategies underscores the absence of consensus in model construction and highlights the need for standardized methodological approaches and external validation to determine stable and transportable predictor sets.

### 3.5. Outcome Definition and Follow-Up Heterogeneity

Definitions of postoperative atrial fibrillation varied considerably across studies. While most defined POAF as new-onset atrial fibrillation occurring during the index hospitalization, duration thresholds differed (e.g., ≥30 s, ≥5 min, or clinically documented episodes requiring intervention). Monitoring strategies also varied, ranging from continuous telemetry to intermittent electrocardiographic assessment or reliance on clinical documentation. Follow-up periods were heterogeneous, with some studies restricting outcomes to in-hospital events and others extending observation to 30 days or beyond. This variability in outcome definition and surveillance intensity likely contributed to differences in reported incidence and model performance.

## 4. Discussion

This systematic review provides a comprehensive appraisal of existing prediction models for postoperative atrial fibrillation after cardiac surgery, highlighting their methodological heterogeneity, predictive performance, and limitations in validation and reporting. Our findings highlight that POAF is a multifactorial complication influenced by demographic, clinical, structural, and biochemical determinants [15]. Advanced age, previous atrial fibrillation, left atrial volume index, hypertension, and systemic inflammation index emerge as the most consistent predictors [17].

The heterogeneity in POAF definitions and follow-up strategies represents an important source of clinical and methodological variability. Differences in monitoring intensity, episode duration thresholds, and follow-up time may influence both outcome incidence and predictor effect estimates. Future model development studies should adopt standardized POAF definitions and clearly report surveillance strategies to enhance comparability and generalizability.

The inclusion of postoperative variables in some models reflects an attempt to construct dynamic risk prediction frameworks rather than purely preoperative stratification tools. While such models may improve short-term predictive accuracy, they may be less suitable for preoperative decision-making. Future research should clearly distinguish between baseline risk stratification models and dynamic perioperative models.

The importance of age as a major determinant of POAF has been confirmed across several studies. In the cohort by Rader et al., age was identified as one of the strongest independent predictors of atrial fibrillation following coronary artery bypass grafting (CABG), particularly among elderly patients, with a higher incidence observed in those aged over 70 years [5,36,43]. This finding aligns with our meta-analysis, where advanced age consistently demonstrated a strong association with POAF. Similarly, Lin et al. reported that older patients had significantly higher rates of POAF and worse long-term outcomes, including increased risk of recurrent atrial fibrillation and cardiovascular mortality [7]. These studies reinforce our pooled analysis showing that age is a fundamental demographic risk factor.

Structural and echocardiographic parameters also proved highly relevant. Our meta-analysis showed the left atrial volume index (LAVi ml/m^2^) was associated with POAF, odds ratio of 1.05. In agreement, Zhang et al. developed and validated a diagnostic model based on left atrial diameter in patients undergoing off-pump CABG, finding that atrial dimensions ≥ 39 mm were independently predictive of POAF and significantly improved risk stratification compared with conventional scores such as CHA_2_DS_2_-VASc [11,20,28,34,41]. Likewise, Lacalzada et al. showed that postoperative echocardiographic assessment of diastolic function and atrial dimensions was highly predictive, with patients displaying diastolic dysfunction grade 2 or 3 having an odds ratio up to 23 for POAF [14,38]. These findings resonate with our results, confirming that echocardiographic indices of atrial structure and function should be central to prognostic evaluation.

Beyond conventional clinical and imaging predictors, molecular and omics-based markers have gained increasing attention [29]. Fischer et al. investigated DNA methylation profiles in patients undergoing cardiac surgery, identifying CpG methylation loci that significantly predicted POAF, with models combining clinical and epigenomic variables achieving an AUC of 0.79 in validation cohorts [13]. Our findings, which emphasize systemic inflammation and biomarkers as strong predictors such as interleukin-6, systemic immune-inflammation index (SII), and metabolic panel predictors [30,31,32], are in line with the concept that epigenetic regulation and inflammatory pathways jointly drive atrial vulnerability [19,25,39]. Similarly, Tan et al. integrated gene expression and methylation data from peripheral blood mononuclear cells, identifying differential expression of genes including WARS2, CKAP2, and CHI3L1 as novel predictors, with their LASSO-based model achieving an AUC of 0.88 in training cohorts [12]. This complements our results by expanding the field from clinical and echocardiographic factors into genomic and transcriptomic risk markers. However, not all inflammatory markers have shown consistent associations; for instance, POAF was not correlated with C-reactive protein in some studies [24].

Further supporting the role of transcriptomics, Gilbers et al. conducted a transcriptomic analysis in cardiac surgery patients and identified circulating gene expression signatures associated with POAF [10]. Although our review found considerable heterogeneity in biomarker studies, the consistent signal across independent investigations supports the emerging role of omics in prediction models. In line with this, Yang et al. applied machine learning techniques to integrate multiple clinical and perioperative predictors, reporting enhanced discriminative ability compared to traditional regression approaches [6,21,22,37]. Our synthesis confirmed that logistic regression remains the most common modeling strategy, but the incorporation of penalized regression and machine learning holds promise for future improvements.

Several studies also provided long-term perspectives. Lin et al. reported that POAF was associated not only with perioperative morbidity but also with long-term recurrence of atrial fibrillation and mortality [7], while Rizza et al. described predictors of subacute POAF during cardiac rehabilitation [40], and Rader et al. noted increased risk of cerebrovascular events in elderly patients with postoperative atrial fibrillation [5]. Similarly, in adults with congenital heart disease, POAF was associated with late-onset atrial fibrillation but not all-cause mortality [23]. These findings parallel our observation that POAF is not a benign, transient complication but a harbinger of poor outcomes, underscoring the need for early identification and prevention strategies.

Taken together, our findings and those of previous literature consistently demonstrate that POAF results from a multifactorial interplay between patient-related, surgical, and molecular factors. Age, left atrial size, systemic inflammation, and genetic or epigenetic regulation all appear to converge in determining susceptibility to postoperative arrhythmogenesis.

### 4.1. Emerging Non-Traditional Predictors and the Cardio-Oncology Interface

In addition to conventional demographic and structural predictors, emerging non-traditional factors deserve particular attention. Cardiopulmonary bypass (CPB) duration has increasingly been recognized as an independent determinant of postoperative atrial fibrillation. Prolonged CPB time amplifies systemic inflammatory activation, oxidative stress, endothelial dysfunction, and atrial structural remodeling, thereby creating a pro-arrhythmogenic substrate. The magnitude of inflammatory response correlates with cytokine release, complement activation, and neurohormonal imbalance, all of which contribute to atrial electrical instability.

Recent evidence has further reinforced this mechanistic framework. Georghiou et al. (2025) [44] demonstrated that active malignancy is an independent determinant of postoperative atrial fibrillation following cardiac surgery, even after adjustment for conventional demographic and surgical risk factors. In their cohort, cancer status remained significantly associated with POAF, suggesting that malignancy-related systemic inflammation, oxidative stress, prothrombotic activation, and autonomic imbalance may enhance atrial vulnerability in the perioperative setting [44].

The incorporation of cardiopulmonary bypass duration, systemic inflammatory burden, and active malignancy into the conceptual framework of POAF significantly strengthens the pathophysiological interpretation of postoperative arrhythmogenesis. These factors converge mechanistically through inflammatory cytokine activation, oxidative stress pathways, endothelial dysfunction, and atrial structural remodeling, thereby reinforcing the hypothesis that POAF is not merely a transient perioperative phenomenon but the expression of a biologically vulnerable atrial substrate.

Expanding the list of independent predictors beyond traditional demographic and structural variables allows a more integrative understanding of perioperative risk. In particular, cancer-related systemic inflammation and extracorporeal circulation-induced immune activation represent clinically measurable contributors that may refine multivariable risk models.

From a clinical perspective, acknowledging these emerging predictors enhances the applicability of risk stratification strategies in high-risk surgical populations, including elderly patients, individuals with active or recent malignancy, and those undergoing prolonged or complex procedures requiring extended cardiopulmonary bypass. Integrating oncologic status and inflammatory markers into perioperative assessment frameworks may improve early identification of vulnerable patients and support tailored monitoring or prophylactic strategies within a multidisciplinary Heart Team approach.

Importantly, the same study also highlighted the contribution of prolonged cardiopulmonary bypass duration as an independent predictor, supporting the hypothesis that extracorporeal circulation acts as an inflammatory amplifier that promotes atrial electrical and structural remodeling.

These findings introduce a clinically relevant cardio-oncology dimension to POAF risk modeling. In contemporary surgical populations—characterized by advanced age and increasing prevalence of cancer survivorship—active malignancy should be considered in perioperative risk stratification frameworks. Integrating oncologic status with inflammatory markers and procedural variables may improve prediction accuracy and align risk assessment with multidisciplinary Heart Team practice.

Systemic inflammatory burden represents another mechanistically relevant domain. Biomarkers such as interleukin-6, neutrophil-to-lymphocyte ratio, and systemic immune-inflammation index reflect perioperative inflammatory stress and have demonstrated independent predictive value across multiple cohorts. These findings support the hypothesis that inflammation-mediated atrial remodeling plays a central role in POAF pathogenesis.

Importantly, recent evidence has introduced a cardio-oncology dimension to POAF risk stratification. Active malignancy appears to be an independent predictor of postoperative atrial fibrillation after cardiac surgery, likely mediated through chronic inflammatory activation, prothrombotic state, endothelial dysfunction, and cancer-related neurohormonal perturbations. This emerging interface between oncology and perioperative cardiology suggests that cancer status should be considered in future risk models, particularly in aging populations undergoing complex cardiac procedures.

Together, CPB duration, systemic inflammatory activation, and active malignancy expand the conceptual framework of POAF beyond traditional clinical predictors and underscore the need for integrative perioperative risk stratification models aligned with contemporary multidisciplinary Heart Team practice.

Active malignancy and prolonged cardiopulmonary bypass (CPB) time should be regarded as independent and multifactorial contributors to postoperative atrial fibrillation. Both factors converge through systemic inflammatory activation, oxidative stress pathways, endothelial dysfunction, and atrial structural vulnerability, thereby amplifying perioperative arrhythmogenic susceptibility. In particular, cancer-related chronic inflammation and CPB-induced immune activation may create a biologically primed atrial substrate that becomes electrically unstable under surgical stress. Future prognostic models should consider integrating oncologic status and procedural inflammatory burden to improve prediction accuracy and better reflect contemporary high-risk surgical populations.

### 4.2. Clinical Implications

Beyond traditional predictors such as age and atrial size, the inclusion of inflammatory burden, cardiopulmonary bypass duration, and oncologic status may refine contemporary perioperative risk stratification models.

From a clinical standpoint, our findings emphasize the importance of incorporating both conventional and novel predictors into perioperative risk assessment, including recently developed tools such as the POLARIS score [27]. Age and atrial size remain central, but biomarkers of inflammation and advanced echocardiographic indices provide additional discriminative value [26]. Molecular markers such as DNA methylation patterns or transcriptomic signatures may further enhance prediction, although their integration into clinical workflows requires validation. Additional biomarkers such as interleukin 6 and systemic immune-inflammation index (SII) have also shown predictive value for POAF [42]. Early identification of high-risk patients could allow for tailored prophylactic strategies, including intensified monitoring, pharmacological prophylaxis in selected cases.

### 4.3. Limitations

Despite the robust evidence synthesized, important limitations must be acknowledged. Firstly, heterogeneity across studies was substantial (I^2^ = 91.9%), reflecting variability in patient populations, surgical techniques, monitoring strategies, and definitions of POAF. Secondly, many studies suffered from methodological limitations, as confirmed by PROBAST, particularly in the analysis domain, including inadequate handling of missing data, lack of shrinkage methods, and insufficient validation. Thirdly, biomarker and omics-based studies, although promising, often had small sample sizes and limited external validation, reducing their immediate clinical applicability. Finally, publication bias cannot be excluded, as negative studies may remain underreported.

This systematic review has several limitations that should be acknowledged. Firstly, considerable heterogeneity existed among the included studies in terms of study design, population characteristics, surgical procedures, and definitions of postoperative atrial fibrillation, which may have influenced the comparability and pooled interpretation of findings. Secondly, most of the prognostic models were developed using retrospective single-center cohorts, often with small sample sizes and without external validation, limiting the generalizability of their predictive performance. Thirdly, important methodological aspects, such as handling of missing data, calibration assessment, and reporting of shrinkage or optimism correction, were inconsistently described, which may have led to model overfitting. Fourthly, publication bias and selective reporting could not be ruled out, as only studies published in English were included. Finally, the absence of patient-level data prevented a meta-analytic synthesis of discrimination and calibration measures across models. Despite these limitations, this review provides the most comprehensive and critical appraisal to date of existing prognostic models for POAF after cardiac surgery.

Although individual predictors were extracted for descriptive purposes, quantitative pooling of heterogeneous predictors was not pursued due to conceptual and methodological heterogeneity across models.

## 5. Conclusions

This systematic review and meta-analysis confirms that POAF is driven by a complex interplay of demographic, structural, biochemical, and molecular predictors. While traditional factors such as age and atrial size remain pivotal, growing evidence highlights the predictive potential of biomarkers and omics-based signatures. However, methodological heterogeneity and lack of validation limit the translation of many models into clinical practice. Future research should prioritize standardized definitions, multicentric validation, and integrative models that combine clinical, imaging, and molecular data to achieve robust, clinically applicable prediction tools.

## Figures and Tables

**Table 1:** Characteristics of included studies.

Author	Year	Design	Sample Size	Candidate Predictors	Final Predictors	Model Type	Internal Validation	External Validation	AUC/C-Statistic	Calibration	EPV
**Rader**	2012 [5]	Prospective cohort	233	Clinical + Echo	3	Logistic regression	Bootstrap	No	0.77	HL + Bootstrap	NR
**Yang**	2022 [6]	Retrospective	422	Clinical + labs	5	Logistic + Nomogram	Bootstrap	No	0.817	Calibration curve	NR
**Lin (PAFAC)**	2018 [7]	Retrospective cohort	762	32 variables	4	Logistic + Score	80/20 split	No	0.60	HL + Lowess	NR
**Helgadottir**	2012 [8]	Retrospective cohort	744	Clinical	4	Logistic	None	No	0.74	Not reported	NR
**Kalisnik**	2019 [9]	Prospective cohort	150	6	3	Logistic	Leave-one-out CV	No	0.804	Not reported	10.3
**Gilbers**	2024 [10]	Prospective cohort	133	Clinical + Biomarkers	Multiple models	Cox models	Time-dependent ROC	No	0.82–0.92	Not reported	NR
**Zhang**	2023 [11]	Retrospective cohort	749	Clinical + echo	7	Logistic + Nomogram	Split validation	No	0.687–0.661	Calibration curves	26.8
**Tan**	2024 [12]	Prospective cohort	139	Clinical + Gene markers	10 (LASSO)	LASSO logistic	5-fold CV + split	No	0.889/0.784	Not reported	4.3
**Fischer**	2022 [13]	Cohort	211	Clinical + CpG	6	Logistic	5-fold CV	Yes	0.83/0.79	Not reported	15
**Lacalzada**	2016 [14]	Cohort	147	Echo variables	3	Logistic	None	No	0.98	HL test	NR
**Ovreiu**	2008 [15]	Cohort	99	ECG	NR	Logistic	None	No	NR	Not reported	NR
**Lu**	2017 [16]	Prospective cohort	126	Hemodynamic	3	Logistic	None	No	NR	Not reported	NR
**Patel**	2018 [17]	Cohort	209	Clinical	Full model	Logistic	None	No	0.76	Not reported	NR
**Candan**	2013 [18]	Prospective	53	Echo strain	3	Logistic	None	No	0.804	Not reported	NR
**Tao**	2024 [19]	Retrospective	102	LAVI + IL-6	2	Logistic	None	No	0.768	ROC only	NR
**Osranek**	2006 [20]	Prospective	205	LAVI	2	Logistic	None	No	0.729	ROC only	NR
**Lu (ML)**	2023 [21]	Prospective	1400	Clinical	Multiple	Logistic + ML	In-sample	No	0.716	Not reported	NR
**Parise**	2023 [22]	Prospective	394	Clinical	NR	Logistic	None	No	NR	Not reported	NR
**Egbe**	2022 [23]	Retrospective	1598	Clinical	NR	Logistic	None	No	NR	Not reported	NR
**Ahlsson**	2007 [24]	Cohort	524	CRP + clinical	NR	Logistic	None	No	NR	Not reported	NR
**Kang**	2018 [25]	Retrospective	442	LAVI	NR	Logistic	None	No	NR	Not reported	NR
**Pollock**	2018 [26]	Multicenter	NR	Existing scores	NR	Logistic	None	No	NR	Not reported	NR
**Rosati (POLARIS)**	2025 [27]	Retrospective	5739	13 predictors	13	Logistic + Score	Bootstrap	Yes (center 2)	0.635	HL *p* = 0.878	Adequate
**Fujiwara**	2014 [28]	Prospective	88	TDI variables	2	Logistic	None	No	0.85	ROC only	17.5
**Vesela**	2023 [29]	Prospective	137	HRV	3	Logistic	None	No	0.86	ROC only	16
**Hinoue**	2023 [30]	Retrospective	212	SII + clinical	3	Logistic	None	No	0.80	ROC only	30
**Qian**	2022 [31]	Retrospective	2974	Electrolytes	NR	Logistic + RF	In-sample	No	0.716	Not reported	NR
**Gu**	2017 [32]	Case–control	100	ECG	3	Logistic	Leave-one-out	No	0.78	ROC only	16.6
**Takashi**	2014 [33]	Retrospective	63	TDI	2	Logistic	None	No	0.737	ROC only	NR
**Takashi**	2016 [34]	Retrospective	73	TDI	2	Logistic	None	No	NR	ROC only	NR
**Zangarillo**	2004 [35]	Prospective	160	Clinical	3	Logistic	None	No	NR	Not reported	NR
**Kievisas**	2017 [36]	Prospective	617	Clinical	NR	Logistic	None	No	NR	Not reported	NR
**Chung**	2023 [37]	Prospective	33,464	Clinical	NR	Logistic	Sensitivity analysis	No	NR	Not reported	NR
**Kouriliouros**	2011 [38]	Prospective	90	Adiponectin	3	Logistic	None	No	NR	Not reported	NR
**Di Gioia**	2017 [39]	Retrospective	134	LAVI	1–2	Logistic	None	No	NR	Not reported	NR
**Rizza**	2023 [40]	Retrospective	737	Clinical	4	Logistic	None	No	0.721	HL *p* = 0.578	NR
**Dalos**	2022 [41]	Retrospective	124	Echo strain	1	Logistic	None	No	NR	NRI/IDI	NR
**Lednev**	2016 [42]	Prospective	39	NT-proBNP	1	Logistic	None	No	0.988	ROC only	NR
**Baker**	2013 [43]	Nested case–control	560	CHA2DS2-VASc	1	Logistic	None	No	NR	Not reported	NR

## Data Availability

The original contributions presented in this study are included in the article/Supplementary Material. Further inquiries can be directed to the corresponding author.

## Supplementary Materials

The following supporting information can be downloaded at: https://www.mdpi.com/article/10.3390/jcm15135255/s1, Supplementary Table S1 (Search strategy). Supplementary Table S2 (PRISMA checklist). Supplementary Material S3 Meta-analysis figures per factor.
